# A Robust and Efficient FRET-Based Assay for Cannabinoid Receptor Ligands Discovery

**DOI:** 10.3390/molecules28248107

**Published:** 2023-12-15

**Authors:** Gemma Navarro, Eddy Sotelo, Iu Raïch, María Isabel Loza, Jose Brea, Maria Majellaro

**Affiliations:** 1 Department of Biochemistry and Physiology, Faculty of Pharmacy and Food Science, University of Barcelona, 08028 Barcelona, Spain; 2 Institute of Neuroscience of the University of Barcelona, 08035 Barcelona, Spain; 3 Department of Organic Chemistry, Center for Research in Biological Chemistry and Molecular Materials (CiQUS), University of Santiago de Compostela, 15782 Santiago de Compostela, Spain; e.sotelo@usc.es; 4 Research Center in Molecular Medicine and Chronic Diseases (CIMUS), University of Santiago de Compostela, 15782 Santiago de Compostela, Spain; mabel.loza@usc.es; 5 Celtarys Research SL, Avda. Mestre Mateo, 2, 15706 Santiago de Compostela, Spain

**Keywords:** CB_1_R, CB_2_R, HTRF, binding, HTS

## Abstract

The identification of new modulators for Cannabinoid Receptors (CBRs) has garnered significant attention in drug discovery over recent years, owing to their manifold pathophysiological implications. In the context of hit identification, the availability of robust and sensitive high-throughput screening assays is essential to enhance the likelihood of success. In this study, we present the development and validation of a Tag-lite^®^ binding assay designed for screening hCB_1_/hCB_2_ binding, employing a dual fluorescent ligand, CELT-335. Representative ligands for CBRs, exhibiting diverse affinity and functional profiles, were utilized as reference compounds to validate the robustness and efficiency of the newly developed Tag-lite^®^ binding assay protocol. The homogeneous format, coupled with the sensitivity and optimal performance of the fluorescent ligand CELT-335, establishes this assay as a viable and reliable method for screening in hit and lead identification campaigns.

## 1. Introduction

Cannabinoid receptors 1 and 2 (CB_1_R and CB_2_R), along with endocannabinoids (endogenous ligands such as 2-arachidonoylglycerol and anandamide) and metabolic enzymes, collectively constitute the Endocannabinoid System (ECS), proven to be significant in both physiological and pathophysiological processes [1]. Patients with Parkinson’s disease exhibit a lower expression of CB_1_R [2] while CB_2_R polymorphisms appear to be associated with conditions such as depression, addiction, and eating disorders [3]. Recent findings also highlight cannabinoids’ ability to impede cancer progression at various stages, exhibiting a synergistic effect when co-administered with anticancer agents. This contributes to antimetastatic and antiangiogenic activities while stimulating immune responses [4].

These insights underscore the promising potential of modulating Cannabinoid Receptors (CBRs) for developing novel chemical entities targeting a range of unmet medical needs. The medicinal chemistry of CBRs, supported by resolved crystal structures of CB_1_R [5] and CB_2_R [6], has led to the identification of several new scaffolds capable of modulating CBR activation at either orthosteric [7] or allosteric sites [8,9].

One of the primary challenges is separating the therapeutic effects derived from CBRs from the side effects, especially psychotropic ones resulting from CB_1_R activation in the central nervous system (CNS). An illustrative case involves Synthetic Cannabinoid Agonists (SCRAs). Pfizer initiated the CP series, including CP5594, in the 1980s as part of a CBR research program aiming to explore their structure-biological activity relationship for developing analgesic drugs [10]. Unfortunately, these compounds exhibited greater potency than THC on CB_1_R in CNS, giving rise to a new class of abused psychoactive substances [11,12].

**Figure 1:**
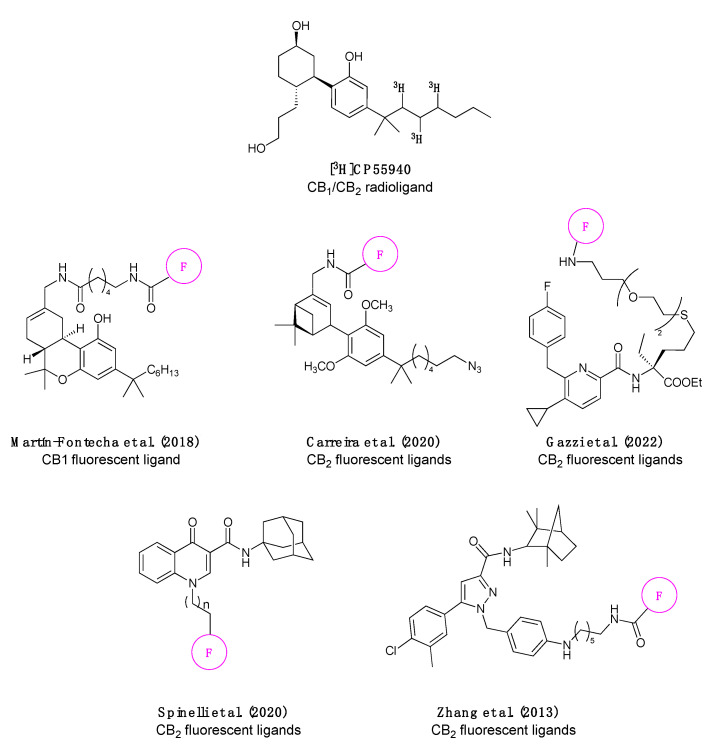
Chemical structure of the most commonly used CB_1_/CB_2_ radioligand [^3^H]CP55940 and the fluorescent ligands published in the literature [14,15,16,17,18] to date (the pink circle bearing the F represents a general fluorophore, since in some publications the same pharmacophore was labelled with multiple fluorophores).

In this context, the need for novel, robust, and cost-effective methodologies for screening compound libraries targeting CB_1_R and CB_2_R becomes imperative for identifying drugs with an optimal clinical profile. Currently, many screening campaigns employ radioactive CP-55940, [^3^H-CP-55940], the initial tool for studying CBR binding (Figure 1) [13]. Efforts have since been made to identify new probes, aiming to replace radioactivity with environmentally friendly methodologies, such as fluorescence (Figure 1).

In the literature, there are instances of CB_1_R-selective fluorescent ligands validated in flow cytometry [14], and CB_2_R-selective fluorescent ligands validated in Time-Resolved Fluorescence Energy Resonance Transfer (TR-FRET) [15,16], as well as receptor visualization in living cells [15,17], mice [18], and zebrafish (Figure 1) [16]. Although many fluorescent probes exhibit specific emission and excitation spectra with considerable intensity, some have demonstrated nonspecific interactions [19].

Homogeneous Time-Resolved Fluorescence (HTRF) is a TR-FRET-based assay [20] conducted in homogeneous conditions, utilizing lanthanide fluorophores such as europium and terbium as donors [21]. Lanthanides offer distinct advantages over conventional fluorophores: their extended fluorescence duration (ranging from several hundred microseconds to a few milliseconds) enables delayed emission readings, and their narrow bands and high Stokes shift prevent cross-excitation and cross-emission phenomena [22]. These features collectively reduce background noise, significantly improving the signal-to-noise ratio (SNR) [22,23] and sensitivity [24]. Lanthanide ions are enclosed in specific complexes (cryptates or chelates), where the cage’s chemical structure can profoundly influence fluorophore properties such as excitation/emission spectra and permeability. Recently developed lanthanide complexes, such as CoraFluors, exhibit enhanced stability and sensitivity, along with a unique membrane permeability, expanding their applicability to target engagement assays in live cells [25].

Labeling strategies for immobilizing donor fluorophores on target proteins have been explored in recent years. Regarding membrane proteins like GPCRs, antibodies have proven unsuitable due to steric hindrance and reverse binding. Consequently, new approaches have been implemented based on suicide enzyme technologies like SNAP-tag [26,27]. SNAP-tag is an engineered mutant of O6-alkylguanine-DNA alkyltransferase (AGT) that is capable of reacting specifically with O6-benzylguanine (BG) derivatives [26]. It has been demonstrated that SNAP-tag allows GPCR labeling with high yields, and due to the small size of this tag, GPCR expression and activity are preserved [26,28].

The Tag-lite^®^ binding assay combines the HTRF detection method with a covalent labeling technology called SNAP-tag^®^. This innovative methodology was initially validated in binding assays for GPCRs, such as chemokine (CXCR4), opioid (δ, μ, and κ), and cholecystokinin (CCK1 and CCK2) receptors [29].

In previous research, CELT-335, a dual hCB_1_/hCB_2_ fluorescent ligand, was validated in the Tag-lite^®^ binding assay to measure CB_1_R binding with three natural CBR ligands [29]. Although excellent dose/response curves were obtained, along with a high correlation with previously published data, the number of reference compounds tested was limited and validation in CB_2_R binding experiments was lacking.

Here, we report the first example of a Tag-lite^®^ binding assay validated for both hCB_1_ and hCB_2_ receptors using a single fluorescent probe, CELT-335. This dual hCB_1_/hCB_2_ fluorescent ligand exhibited high specific binding (signal-to-noise ratio) and FRET signal, making it suitable for screening compounds targeting both cannabinoid receptor subtypes. The CB_1_/CB_2_ dual activity of the fluorescent ligand CELT-335, combined with the advantageous Tag-lite^®^ binding assay technology, provides a reliable, robust, and cost-effective alternative for CB_1_R and CB_2_R screening campaigns.

## 2. Results

**Figure 2:**
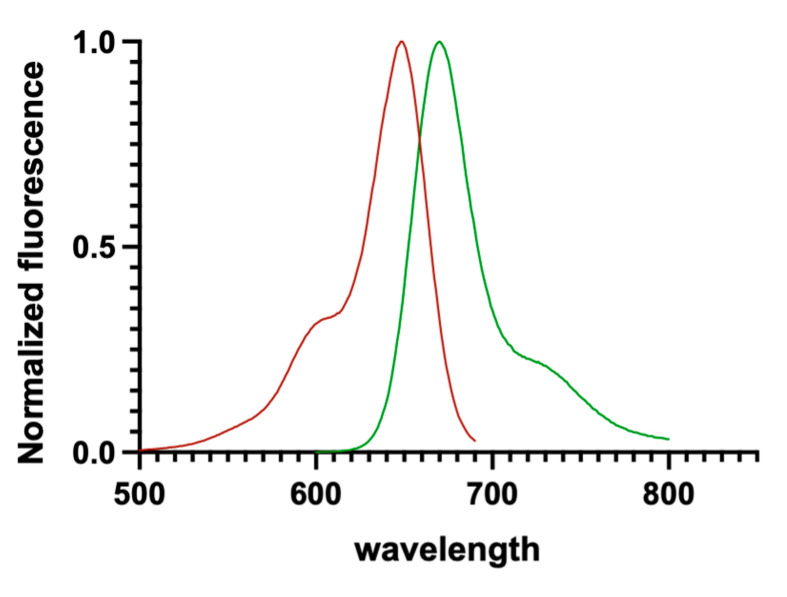
Excitation/emission spectra of CELT-335 measured in MeOH. Excitation spectra is represented in red, emission spectra in green.

The workflow comprised three main steps: the development of a fluorescent ligand suitable for CB_1_/CB_2_ receptors in Tag-lite^®^ binding assays, characterization of the identified fluorescent ligand (CELT-335), and its validation as a tool for the specific assay of interest. The primary parameters considered to select the optimal fluorescent ligand were its affinity for the CBRs and its photophysical properties. As depicted in Figure 2, CELT-335 exhibits λexc and λem at 650 nm and 673 nm, respectively. These wavelengths are compatible with the emission spectra of Terbium [24], the lanthanide used for CBRs labeling in the developed Tag-lite^®^ binding assay.

### 2.1. CELT-335 Binding at CB_1_ and CB_2_ Receptors

The binding affinity of the CELT-335 fluorescent ligand was assessed through radioligand binding assays, revealing a nanomolar affinity for both CB_1_ and CB_2_ receptors (see Table 1). The ligand exhibited a 6-fold higher affinity for CB_2_R in the radioligand binding assay (Table 1). Encouraged by these initial promising findings, saturation experiments were conducted using the Tag-lite^®^ binding assay for both CB_1_ and CB_2_ receptors, demonstrating K_d_ values comparable to those obtained previously via radioligand binding assays.

**Figure 3:**
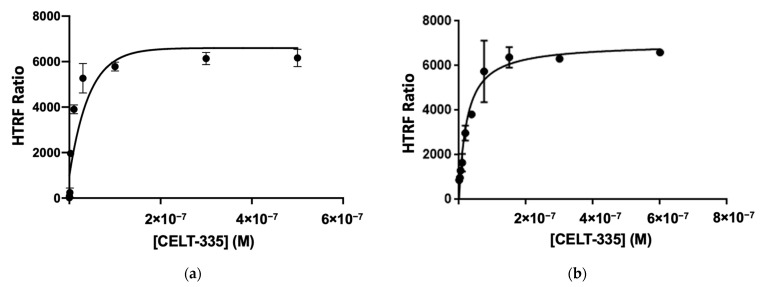
Saturation assays using CELT-335. Specific binding is shown, obtained from total binding and unspecific binding (**a**) CB_1_R expressing adherent HEK-293T cells and unspecific binding measurement (specific binding measured using CP55490 at 10 μM concentration) (**b**) CB_2_R expressing adherent HEK-293T cells and unspecific binding measurement (specific binding measured using GW405833 at 10 μM concentration). Data represent the mean ± SEM (n = 3 in triplicates).

In these saturation experiments, specific binding to CB_1_ and CB_2_ receptors was investigated, employing appropriate competitors: CP55490 at 10 µM for CB_1_R and GW405833 at 10 μM for CB_2_R (see Figure 3). The high affinity of CELT-335 for CB_1_R (Ki= 44.8 nM) observed in radioligand binding was preserved in the Tag-lite^®^ saturation binding assay in HEK-293 T cells expressing SNAP-CB_1_R (K_d_ = 42 nM), yielding an excellent HTRF signal (HTRF Ratio 665/620, see Figure 3).

### 2.2. CELT-335 HTRF Assay Validation in hCB_1_R Expressing Adherent Cells

**Figure 4:**
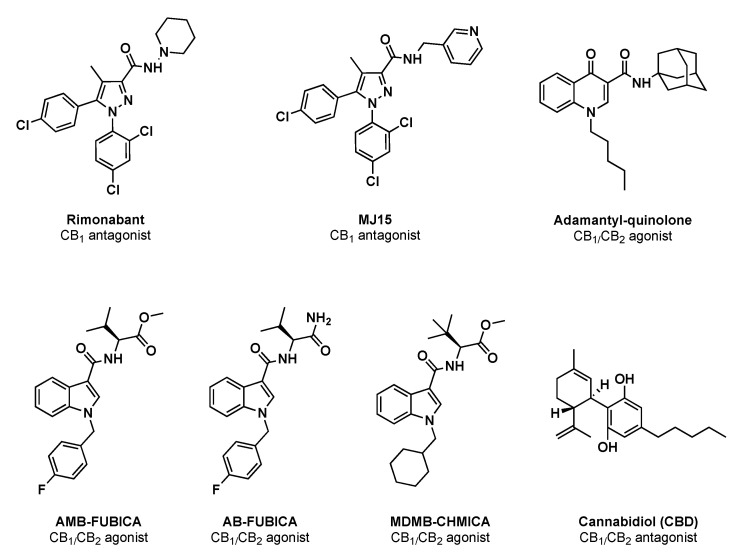
Chemical structures and functional activity of the reference compounds used for CELT-335 validation in Taglite^®^ binding assays.

To validate the potential of CELT-335 as a fluorescent probe for hCB_1_/CB_2_ receptors in library screening, a set of seven well-known reference compounds was selected. As shown in Table 2 and Figure 4, these selected CBRs ligands encompass different chemical scaffolds and exhibit diverse affinity, selectivity, and functional activities, enabling appropriate validation.

**Figure 5:**
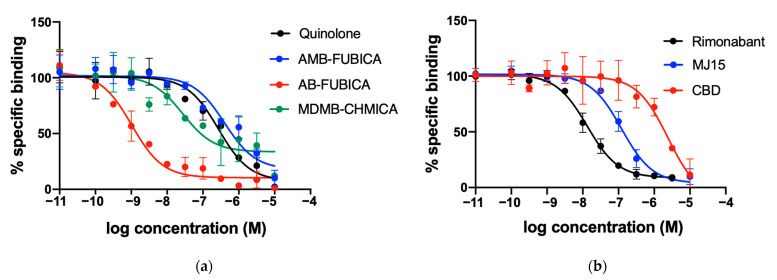
Competition experiments of binding of fluorescent ligand CELT-335 to living HEK-293 T cells expressing the SNAP-CB_1_R. Tb labelling was performed as described in Materials and Methods. Tag-Lite^®^ competition binding curves were obtained by using 100 nM of CELT-335 and increasing concentrations of compounds tested (0–10 μM). HTRF Ratio = 665 nm acceptor signal/620 nm donor signal × 10,000; the percentage is calculated by taking the highest value as 100%. Data represent the mean ± SEM (n = 5 in triplicates). (**a**) concentration/response curves obtained for agonists; (**b**) concentration/response curves obtained for the antagonists.

As mentioned earlier, the high affinity of CELT-335 for CB_1_R (K_i_ = 44.8 nM) observed in radioligand binding was preserved in the Tag-lite^®^ saturation binding assay in HEK-293 T cells expressing SNAP-CB_1_R (K_d_ = 42 nM), yielding an excellent HTRF signal (HTRF Ratio 665/620, Figure 3). These results served as a starting point for the development of the Tag-lite^®^ binding CB_1_R binding assay. As observed in Figure 5, coherent and well-defined sigmoidal concentration/response curves were obtained for the seven reference compounds tested, and the corresponding pKi values were calculated and compared with the data previously reported in the literature (see Table 2).

### 2.3. CELT-335 HTRF Assay Validation in hCB_2_R Expressing Adherent Cells

**Figure 6:**
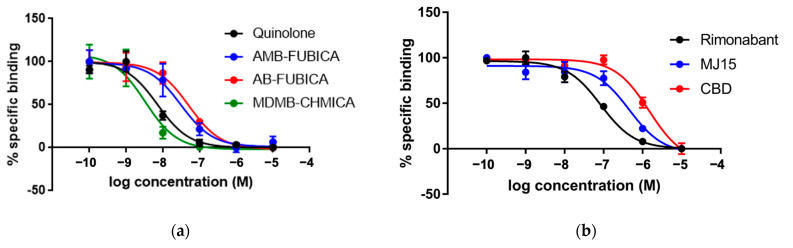
Competition experiments of binding of fluorescent ligand CELT-335 to living HEK-293 T cells expressing the SNAP-CB_2_R. Tb labelling was performed as described in Materials and Methods. Competition binding curves were obtained through Tag-lite^®^ technology using 10 nM of CELT-335 and increasing concentrations of compounds tested (0–10 μM). HTRF Ratio = 665 nm acceptor signal/620 nm donor signal × 10,000; the percentage is calculated by taking the highest value as 100%. Data represent the mean ± SEM (n = 5 in triplicates). (**a**) concentration/response curves obtained for agonists; (**b**) concentration/response curves obtained for the antagonists.

As in the case of CB_1_R, the K_d_ obtained through the Tag-lite^®^ saturation binding assay (24.2 nM) confirmed the previously observed affinity of CELT-335 for CB_2_R and demonstrated high FRET between Terbium and the fluorescent ligand (HTRF signal, Ratio 665/620, see Figure 3). Subsequent competition binding assays, conducted with the same set of reference compounds used in the Tag-lite^®^ CB_1_R competition binding experiments, exhibited an excellent correlation with previously reported data (see Table 3) and optimal sigmoidal shapes in concentration/response curves (see Figure 6).

## 3. Discussion

**Figure 7:**
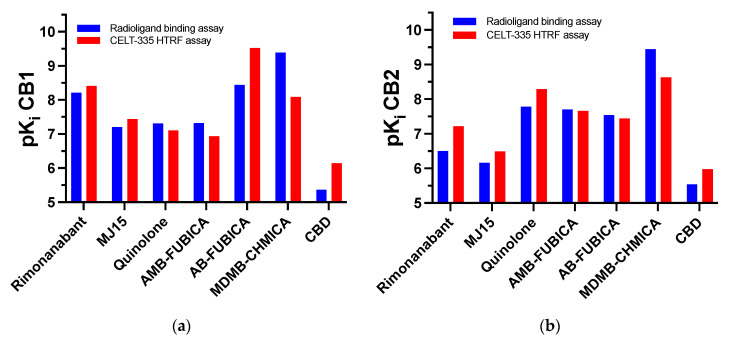
Schematic representation of the high correlation between reference compounds affinity data obtained through radioligand binding assays and the Tag-lite^®^ binding assay developed in this work. (**a**) CB_1_R binding affinities; (**b**) CB_2_R binding affinities.

The saturation experiments conducted with CELT-335 revealed a 2-fold difference in K_d_ for hCB_1_ and hCB_2_ receptors, respectively, along with an excellent FRET signal. High specific binding was observed, measured by adding 10 μM concentrations of appropriate competitors (CP55490 for CB_1_R and GW405833 for CB_2_R). During assay optimization, based on the K_d_ values obtained from saturation studies, the probe concentrations employed for competition studies were set at 100 nM and 10 nM for CB_1_ and CB_2_, respectively. The differing affinity of CELT-335 for CB_1_R and CB_2_R receptors guided the identification of the probe concentrations to be used. Subsequent Tag-lite^®^ binding assays performed with CELT-335 produced reproducible sigmoidal concentration/response curves and affinity data (Ki) with a very high correlation to those obtained through radioligand binding assays (see Figure 7).

**Figure 8:**
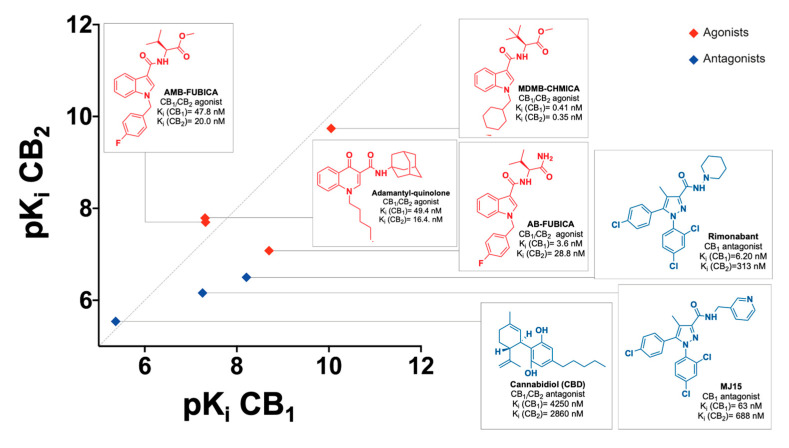
Chemical structures, selectivity (radioligand binding assays K_i_), and functional activity (blue for antagonists and red for agonists) of the reference compounds used for CELT-335 validation in Tag-lite^®^ competition binding assays.

The set of CBRs reference ligands for assay validation was carefully chosen to encompass the highest diversification in terms of affinity, selectivity, functional activity, and chemical structure. A useful graphic of ligands’ affinity and selectivity is presented in Figure 8.

CELT-335 demonstrated exquisite competition with both synthetic and naturally derived cannabinoid ligands, irrespective of their functional activity (agonists or antagonists). It effectively discriminated compounds with very high affinity, such as SCRAs (AMB-FUBICA, AB-FUBICA, MDMB-CHMICA), as well as ligands with medium and low affinity ranges, like Rimonabant and MJ15, or the adamantyl-quinoline.

Since none of the compounds exhibited a difference greater than 1 between the pKi values obtained in the two different experiments (radioligand binding and Tag-lite^®^ binding), as depicted in Figure 8, the validation of CELT-335 in Tag-lite^®^ competition binding assays was deemed successful. A broader range of reference compounds will be screened for further validation, including known screening chemical libraries composed of compounds with diverse chemical structures and targets beyond CBRs.

## 4. Materials and Methods

### 4.1. CELT-335 and Reference Compounds Used for Assay Validation

The commercially available hCB_1_/CB_2_Rs fluorescent ligand CELT-335 was provided by Celtarys Research, while the reference compounds used for assay validation were synthesized by us following previously published experimental procedures [32,33,34,35] except for MJ15 and CBD, which were purchased by TOCRIS and Sigma Aldrich, respectively.

All starting materials, reagents, and solvents used in the synthesis were purchased and used without further purification. After extraction from aqueous phases, the organic solvents were dried over anhydrous magnesium sulfate. The reactions were monitored by TLC on 2.5 mm Merck silica gel GF 254 strips, and the purified compounds each showed a single spot. Unless stated otherwise, UV light and/or iodine vapor were used to detect compounds. The purity and identity of all tested compounds were established through a combination of HPLC, mass spectrometry, and NMR spectroscopy. Purification of isolated products was carried out through column chromatography (Kieselgel 0.040−0.063 mm, E. Merck) or medium-pressure liquid chromatography (MPLC) on a Combi Flash Companion (Teledyne ISCO) with RediSep pre-packed normal-phase silica gel (35−60 μm) columns followed by recrystallization. The NMR spectra were recorded on Bruker AM300 and XM500 spectrometers. Mass spectra were obtained on a Varian MAT 711 instrument.

The excitation and emission spectra of CELT-335 were measured in methanol, using an Edinburgh FS5 spectrofluorometer equipped with a standard cuvette holder (SC5).

### 4.2. Radioligand Binding Assay

#### 4.2.1. Competition Binding in Human CB_1_R

CB_1_R competition binding experiments were carried out by employing cell membranes obtained from a stable CHO-hCB_1_ cell line. Cell membranes were prepared by washing the cells twice with phosphate buffered saline (PBS) and scraped from the plate in lysis buffer (5 mM Tris-HCl, 2 mM EDTA, pH = 7.4). Cell suspension was homogenized and centrifuged at 1000× *g* for 10 min at 4 °C. Supernatant was isolated and centrifuged at 48,000× *g* for 1 h at 4 °C. The membrane’s pellet was suspended in a storage buffer (50 mM Tris-HCl, pH = 7.4). The protein concentration of the cell membrane suspension was 4292 µg/mL.

Radioligand binding assays were performed in a polypropylene 96-well plate containing 20 μg of cell membranes, 1.25 nM [^3^H]-CP55940 (101 Ci/mmol, 1 mCi/mL, Perkin Elmer NET1051250UC), and the compounds under study. Non-specific binding was determined in the presence of Surinabant 10 μM. The reaction mixture (Vt: 250 μL/well) was incubated at 30 °C for 60 min, 200 μL was transferred to a GF/B 96-well plate (Millipore, Madrid, Spain) and treated with binding buffer (50 mM Tris-HCl, 5 mM MgCl_2_, 1 mM EDTA, 0.5% BSA. pH: 7.4); afterward, it was filtered and washed four times with 250 μL wash buffer (50 mM Tris-HCl, 5 mM MgCl_2_, 1 mM EDTA, 0.5% BSA. pH: 7.4), before its measurement in a microplate beta scintillation counter (Microbeta Trilux, PerkinElmer, Madrid, Spain).

#### 4.2.2. Competition Binding in Human CB_2_R

CB_2_R competition binding experiments were carried out by employing cell membranes obtained from a stable HEK-hCB_2_ cell line. Cell membranes were prepared by washing the cells twice in PBS and scraped from the plate in PBS. Cell suspension was centrifuged at 1500× *g* for 3 min at 4 °C. Cell pellet was suspended in lysis buffer (15 mM Tris-HCl, 2 mM MgCl_2_, 0.3 mM EDTA, 1 mM EGTA, pH = 7.5), homogenized, and centrifuged at 40,000× *g* for 25 min at 4 °C. Pellet was suspended in lysis buffer and centrifuged at 40,000× *g* for 1 h at 4 °C. The membrane’s pellet was suspended in storage buffer (7.5 mM Tris-HCl, 12.5 mM MgCl_2_, 0.3 mM EDTA, 1 mM EGTA, 250 mM sucrose, pH = 7.5). The protein concentration of the cell membrane suspension was 4781 µg/mL.

Radioligand binding assays were performed in a polypropylene 96-well plate containing 30 μg of membranes, nM [^3^H]-CP55940 (101 Ci/mmol, 1 mCi/mL, Perkin Elmer NET1051250UC), and the compounds under study. Non-specific binding was determined in the presence of GW405833 10 μM (Sigma G1421). The reaction mixture (Vt: 250 μL/well) was incubated at 30 °C for 90 min, 200 μL was transferred to GF/C 96-well plate (Millipore, Madrid, Spain), pre-treated with 0.5% of PEI, and treated with binding buffer (50mM Tris-HCl, 5mM MgCl_2_, 2.5 mM EGTA, 0.1% BSA. pH: 7.4); afterward, it was filtered and washed four times with 250 μL wash buffer (50mM Tris-HCl, 5mM MgCl_2_, 2.5 mM EGTA, 1% BSA. pH: 7.4), before its measurement in a microplate beta scintillation counter (Microbeta Trilux, PerkinElmer, Madrid, Spain).

### 4.3. Tag-Lite^®^ Saturation and Competition Binding Assays

#### 4.3.1. Statistical Analysis and Curve Fitting

All the experiments were carried out in triplicate. Data from saturation studies were fitted to a one site specific binding by using GraphPad Prism (v 7.00). Data from competition studies were fitted to a four-parameter logistic curve by using GraphPad Prism (v 7.00). K_i_ values were calculated by using the equation K_i_ = IC_50_/(1 + (R/K_d_)), where IC_50_ is the concentration that inhibits the specific binding by 50%, R is the concentration of the fluorophore added to each well, and K_d_ is the dissociation constant derived from saturation studies.

#### 4.3.2. Tag-Lite^®^ Saturation and Competition Binding Assays in Living Cells Expressing CB_1_R

##### Expression Vector

cDNAs for the human version of cannabinoid CB_1_R without their stop codon were obtained through PCR and subcloned to a SNAP-containing vector (Tag-Lite SNAP(+) plasmid (Revvity) using sense and antisense primers harboring unique restriction sites for HindIII and BamHI generating the SNAP tagged CB_1_R (CB_1_R-SNAP).

##### Cell Culture and Transfection

HEK 293T cells were grown in DMEM supplemented with 2 mM L-glutamine, 1 mM sodium pyruvate, 100 units/mL penicillin/streptomycin, and 5% (*v*/*v*) FBS [all supplements were from Invitrogen, (Paisley, Scotland, UK)].

Cells were maintained at 37 °C in a humidified atmosphere of 5% CO_2_ and were passaged with enzyme-free cell dissociation buffer (13151-014, Gibco R, Thermo Fisher, Waltham, MA, USA) when they were 80–90% confluent, i.e., approximately twice per week. Cells were transiently transfected with the PEI (Polyethylenimine, Sigma, St. Louis, MO, USA) method, as previously described [36]. Briefly, HEK 293 T cells were incubated for 4 h with the corresponding cDNA together with polyethyleneimine (5 µL/g cDNA of 10 M polyethyleneimine) and 150 mM NaCl in a serum-free medium. After 4 h, the medium was changed to a fresh complete culture medium. Forty-eight hours after transfection, the cells were washed twice in quick succession in HBSS with 10 mM glucose, detached, and resuspended in the experimental buffer.

##### Labeling of Cells Expressing SNAP-Tagged CB_1_R

The cell culture medium was removed from the 25 cm^2^ flask, and 100 nM SNAP-Lumi4-Tb labeling reagent (Revvity), previously diluted in 3 mL of Tag-lite Buffer (Revvity) 1×, was added to the flask and incubated for 1 h at 37 °C under 5% CO_2_ atmosphere in a cell incubator. The cells were then washed four times with 2 mL of Tag-lite Buffer 1× to remove the excess of SNAP-Lumi4-Tb, detached with enzyme-free cell dissociation buffer, centrifuged for 5 min at 1500 rpm, and collected in 1 mL of Tag-lite Buffer 1×. Tag-lite-based binding assays were performed 48 h after transfection. Densities in the 2500–3000 cells/well range were used to carry out binding assays in white opaque 384-well plates.

##### Competition and Saturation Binding Assays

For competition and saturation binding assays, CELT-335 and the test ligand were diluted in Tag-lite Buffer (TLB) 1×. For competition assays, HEK-293T cells transiently expressing Tb-labeled SNAP-CB_1_R were incubated with 100 nM CELT-335 in the presence of increasing concentrations (0–10 µM range) of test ligand. For saturation assays, HEK-293T cells transiently expressing Tb-labeled SNAP-CB_1_R were incubated with increasing concentrations of CELT-335. Plates contained 10 µL of labeled cells and 5 µL of TLB 1× or 5 µL of the test ligand dilution were added prior to the addition of 5 µL of CELT-335. The plates were then incubated for at least 2 h at room temperature before signal detection. The signal was detected using an EnVision microplate reader (PerkinElmer, Waltham, MA, USA) equipped with a FRET optic module allowing donor excitation at 337 nm and signal collection at both 665 and 620 nm. A frequency of 10 flashes/well was selected for the xenon flash lamp excitation. The signal was collected at both 665 and 620 nm using the following time-resolved settings: delay, 150 ms; integration time, 500 ms. HTRF Ratios were obtained by dividing the acceptor (665 nm) by the donor (620 nm) signals and multiplying by 10,000. The 10,000-multiplying factor was used solely for the purpose of easier data handling.

#### 4.3.3. Tag-Lite^®^ Saturation and Competition Binding Assays in Living Cells Expressing CB_2_R

##### Expression Vector

SNAP-Tag CB_2_R cloned plasmid was provided by Revvity (custom reagent). It is encoding SNAP-Tag at the N-terminal position of human CB_2_R (within Tag-Lite SNAP(+) vector).

##### Cell Culture and Transient Transfection

HEK-293 cells were grown in an MEM medium supplemented with 10% FBS, 1% penicillin-streptomycin, 1% non-essential aminoacids, and 1 mM sodium pyruvate for 75 cm^2^ flask until being 80% confluent. Then they were transiently transfected with 3 µg/mL of SNAP-tagged CB_2_ plasmid for 24 h (37 °C, 5%CO_2_) with lipofectamine 2000 (Invitrogen). Then, 10^5^ cells/well were seeded in a 96-well white plate (Greiner, Kremsmünster, Austria) pre-treated for 30 min with poly-l-ornythine (Sigma) and incubated for 24 h under the same conditions of temperature and CO_2_.

##### Labeling of Cells Expressing SNAP-Tagged CB_2_R

The cell culture medium was removed from the wells, and 600 nM SNAP-Lumi4-Tb, previously diluted in 3 mL of TLB 1×, was added to each well (100 µL) and incubated for 1 h at 37 °C under 5% CO_2_ atmosphere in a cell incubator.

##### Competition and Saturation Binding Assays

The cells were washed with TLB four times, and 50 µL of TTLB were added to each well. In competition assays, 25 µL of test ligand dilution (4x final concentration) were added to each well, while in saturation assays, 25 µL of TLB were added to the wells. A total of 25 µL of CELT-335 at different concentrations were added to each well in saturation assays, and at 40 nM (4-fold the final concentration) in competition assays. The plate was incubated at 22 °C for 1 h. The HTRF signal was detected in a Genius M1000Pro (Tecan, Männedorf Switzerland). A frequency of 10 flashes/well was selected for the xenon flash lamp excitation at a wavelength of 337 nm. The signal was collected at both 665 and 620 nm using the following time-resolved settings: delay, 200 ms; integration time, 500 ms. The HTRF Ratios were obtained by dividing the acceptor (665 nm) by the donor (620 nm) signals and multiplying by 10,000. The 10,000-multiplying factor was used solely for the purpose of easier data handling.

## 5. Conclusions

CBRs have been extensively studied and characterized as targets for treating various diseases over the last few decades [37,38]. Currently, structural analogues of natural cannabinoids are the only drugs employed in clinics, primarily for managing the nausea and vomiting associated with chemotherapy [39,40] and multiple sclerosis [41]. In an effort to advance drug research in this field, we validated a dual hCB_1_/hCB_2_Rs fluorescent ligand (CELT-335) in the Tag-lite^®^ competition binding assay, demonstrating its suitability for screening new compounds with diverse chemical structures, functional activities, and ranges of affinity for both subtypes of cannabinoid receptors.

Despite some drawbacks, such as the relatively high cost of reagents, particularly the lanthanides used as donor fluorophores, and the need for covalent labelling of the target, introducing an additional step to the cell preparation protocol, the aforementioned advantages of Tag-lite^®^ technology and the high specific interaction and affinity of CELT-335 for cannabinoid receptors effectively mitigate these limitations.

CELT-335 shows promise for application in further experiments, including receptor expression studies and fluorescence microscopy. It provides a robust, reliable, and cost-effective methodology to advance the characterization of cannabinoid receptors through fluorescence-based assays.

New development projects are currently underway in our laboratories, focusing on identifying modulators of CBRs and their fluorescent conjugates. This initiative aims to provide tools suitable for various fluorescence-based assays and to broaden the range of probes for both compound screening and the pathophysiological characterization of human CBRs.

## Figures and Tables

**Table 1:** Comparison of CELT-335 affinity for CB_1_ and CB_2_ receptors measured by competition radioligand binding assay (Ki) and saturation Tag-lite^®^ binding assay (K_d_).

	pK_i_^1^		pK_d_^2^	
	CB_1_^3^	CB_2_^4^	CB_1_^5^	CB_2_^6^
CELT-335	7.34 ± 0.11	8.13 ± 0.09	7.37 ± 0.06	7.62 ± 0.04

^1^ Competition radioligand binding assay. ^2^ Saturation assay by Tag-lite^®^ binding assay. ^3^ Displacement of specific [^3^H]-CP55940 binding in human HEK-CB_1_ cells expressed as K*_i_* ± SEM in nM (n = 3) or percentage displacement of specific binding at a concentration of 1 μM (n = 2). ^4^ Displacement of specific [^3^H]-CP55940 binding in human HEK-CB_2_ cells expressed as pK*_i_* ± SEM in nM (n = 3) or percentage displacement of specific binding at a concentration of 1 μM (n = 2). ^5^ pK_d_ calculated through saturation of CELT-335 in human HEK-293T cells transiently expressing Tb-labeled SNAP-CB_1_R. ^6^ K_d_ calculated through saturation of CELT-335 in human HEK-293T cells transiently expressing Tb-labeled SNAP-CB_2_R.

**Table 2:** Comparison of affinity data for CB_1_ receptor of the set of reference compounds obtained through the radioligand competition binding assay and CB_1_ competition binding assays with Tag-lite^®^ technology using CELT-335.

Compound	Functional Activity	pK_i_ CB_1_	
		Radioligand Binding	Tag-Lite^®^ Binding (CELT-335)
Rimonabant	CB_1_ Antagonist	8.21 [30]	8.41 ± 0.07
MJ15	CB_1_ Antagonist	7.20 ± 0.12	7.44 ± 0.08
Adamantyl-quinolone	CB_1_/CB_2_ agonist	7.31 ± 0.09	7.10 ± 0.08
AMB-FUBICA	CB_1_/CB_2_ agonist	7.32 ± 0.07	6.93 ± 0.13
AB-FUBICA	CB_1_/CB_2_ agonist	8.44 ± 0.11	9.52 ± 0.09
MDMB-CHMICA	CB_1_/CB_2_ agonist	9.38 [31]	8.09 ± 0.11
CBD	CB_1_/CB_2_ Antagonist	5.37 [29]	6.15 ± 0.13

Values represent the mean ± SEM of triplicate determinations. Reference is not indicated for those compounds whose affinity through the radioligand binding assay was measured experimentally following published protocols [17].

**Table 3:** Comparison of affinity data for hCB_2_R of a set of reference compounds obtained through the radioligand competition binding assay and hCB_2_R competition binding assays with Tag-lite^®^ technology using CELT-335.

Compound	Functional Activity	pK_i_ CB_2_	
		Radioligand Binding	Tag-Lite^®^ Binding (CELT-335)
Rimonabant	CB_1_ Antagonist	6.50 [30]	7.22 ± 0.06
MJ15	CB_1_ Antagonist	6.16 ± 0.07	6.49 ± 0.12
Adamantyl-quinolone	CB_1_/CB_2_ agonist	7.78 [32]	8.29 ± 0.11
AMB-FUBICA	CB_1_/CB_2_ agonist	7.70 ± 0.08	7.66 ± 0.13
AB-FUBICA	CB_1_/CB_2_ agonist	7.54 ± 0.07	7.44 ± 0.11
MDMB-CHMICA	CB_1_/CB_2_ agonist	9.45 [30]	8.63 ± 0.15
CBD	CB_1_/CB_2_ Antagonist	5.54 [30]	5.98 ± 0.08

Values represent the mean ± SEM of triplicate determinations. Reference is not indicated for those compounds whose affinity through the radioligand binding assay was measured experimentally following published protocols [17].

## Data Availability

Data are contained within the article.
